# Development of a Zynq-Based Seismic Acquisition Station for the Exploration of Antarctic Subglacial Lakes

**DOI:** 10.3390/s24237667

**Published:** 2024-11-30

**Authors:** Keyu Zhou, Qisheng Zhang, Linyan Guo, Guangkun Feng, Changhong Li, Jinhang Zhang, Qifei Zhang

**Affiliations:** 1 School of Geophysics and Information Technology, China University of Geosciences (Beijing), Beijing 100083, China; keyuzhou@email.cugb.edu.cn (K.Z.); guoly@cugb.edu.cn (L.G.); 3010230014@email.cugb.edu.cn (J.Z.); qifeizhang@email.cugb.edu.cn (Q.Z.); 2 School of Instrumentation and Optoelectronic Engineering, Beihang University, Beijing 100191, China; fenggk@buaa.edu.cn; 3 School of Electrical and Electronic Engineering, Trinity College Dublin, Dublin 2, D02 VF25 Dublin, Ireland; li.changhong@postgrad.wit.ie

**Keywords:** Antarctic subglacial lakes, seismic acquisition station, Zynq

## Abstract

The Antarctic region holds significant scientific research value and potential resources. Currently, limited research exists on the use of seismic exploration methods for Antarctic subglacial lakes compared to their use on other continents. Moreover, few reports are available on systems capable of multi-channel seismic data acquisition, remote data quality monitoring, and high-speed real-time data recycling in the extremely low temperatures of Antarctica. In this study, we developed a Zynq-based seismic acquisition station for polar exploration. The system features a compact design, lightweight construction, high data collection accuracy, excellent cold resistance, low power consumption, and real-time control. The software and hardware design of the system are described here, and validity testing is presented. The main controller utilizes a Zynq series system-on-chip integrated with an FPGA (Field-Programmable Gate Array) and an ARM (Advanced RISC Machine), enabling functions such as local data storage on a secure digital card, Wi-Fi wireless human–machine interaction, and high-speed Ethernet data transmission. Furthermore, to enhance data acquisition accuracy under low-temperature conditions, a neural network was employed for the temperature drift correction of the analog-to-digital converter chip. The validity test results showed that the station operated stably, was easy to use, and met the high-standard requirements for polar exploration.

## 1. Introduction

The Antarctic region has high scientific research value and abundant resources and energy. It hosts the world’s largest iron ore and coal reserves, abundant marine life, significant phosphorus and hydrocarbon resources, and over 72% of the Earth’s natural freshwater resources [1,2,3]. Despite its scientific significance, the Antarctic region remains a blind spot in earth sciences.

Acting as a natural laboratory for investigating Earth’s evolutionary history and predicting its future, the Antarctic region features subglacial lakes at the ice sheet’s base, between the ice and subglacial strata. Accessing samples from these subglacial lakes holds the promise of answering questions about the dynamics of the Antarctic ice sheet and the characteristics of ancient organisms [4]. Since their discovery, the in-depth investigation of Antarctic subglacial lakes has become a major focal point in Antarctic exploration [5,6,7]. These subglacial lakes are vital for studying large-scale dynamic changes in the global climate. They may contain records of global climate changes, making them essential for researching future climate change and global sea level rise [8,9,10].

**Figure 1:**
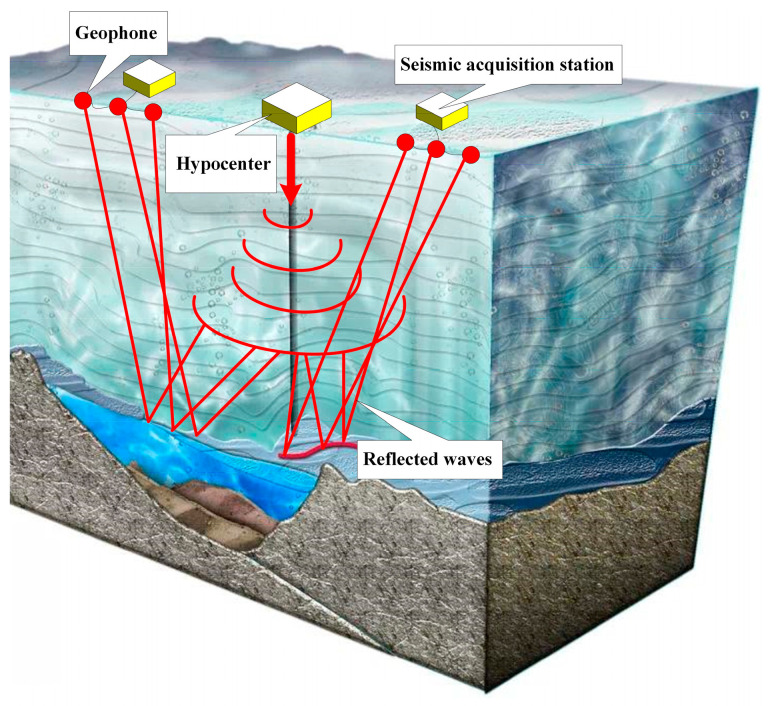
Illustration of seismic exploration of subglacial lakes.

With the rapid advancement in electronic, computer, and data processing technologies, artificial seismic exploration methods have seen significant progress due to their high resolution [11,12,13,14]. Seismic exploration is currently one of the most important, effective, and widely applied methods in geophysical exploration. As illustrated in Figure 1, the seismic exploration of Antarctic subglacial lakes is based on seismic wave field theory, which analyzes the physical morphologies and chemical properties of the target geological strata by utilizing information carried by elastic waves that have been artificially excited and reflected or refracted through different rock strata [15].

Artificial seismic exploration offers high accuracy and resolution, with current capabilities of reaching depths ranging from several dozen meters to several dozen kilometers. It is characterized by the capability for all generated vibration frequencies to propagate through geological strata, good energy conservation, and minimal damage to rocks and the ground surface. Moreover, it exhibits a strong anti-interference capability and a high signal-to-noise ratio [16]. Artificial seismicity has wide applications in seismic exploration, active fault detection [17,18], nuclear explosion detection [19], and volcanic activity detection [20]. It overcomes the limitations of natural seismicity, such as uneven spatial distribution and unknown seismic sources, presenting promising application prospects. In addition, artificially induced seismic waves can aid in observing subsurface rock structures and configurations.

Since the Antarctic’s topographical structure is relatively uniform, primarily consisting of ice and snow, seismic waves can penetrate to greater depths in the Antarctic region than on other continents. Therefore, artificial seismic exploration is one of the most feasible and effective methods for studying Antarctic subglacial lakes. Consequently, developing seismic acquisition systems suitable for polar applications is of substantial significance.

The SUMMIT X One, developed by the German company DMT, is a popular seismic acquisition station with a dynamic range of up to 132 dB at a 2 ms sampling interval. However, its operating temperature is limited to only −25 °C to +60 °C. Other stations, including Geospace Technologies’ GSX, GSB, and GCL seismic acquisition stations, have a dynamic range of 124 dB at a 2 ms sample interval and a synchronization accuracy of <1 μs. SmartSolo, developed by DTCC, has been used in the Antarctic. Still, the instrument can only store data locally, and the system cannot achieve real-time data recycling or allow for remote instrument monitoring.

In this study, we designed a Zynq-based seismic acquisition station to explore Antarctic subglacial lakes capable of multi-channel seismic data acquisition, remote data quality monitoring, and high-speed, real-time data recycling.

## 2. Materials and Methods

### 2.1. Overall Design of Zynq-Based Seismic Acquisition Station for Antarctic Subglacial Lakes

The primary focus of this study was the investigation of seismic data acquisition techniques and the development of a distributed, high-accuracy seismic acquisition station designed for polar applications. The station was designed to withstand the extreme cold temperatures typical of polar regions and is capable of local data storage and wireless, real-time data transmission. Owing to the challenging natural conditions in Antarctica that impede equipment maintenance, minimizing power loss to prolong the station’s operational time and reduce manual intervention was crucial. The overall design of the station, including the chip selection, circuit design, and software control, prioritized low-temperature resilience and power optimization. By adopting a low-power design philosophy, the station aims to minimize inactive power consumption, significantly enhancing its operational duration in severe conditions with a given energy supply. To further reduce the power consumption, a power board was integrated to prevent unnecessary power loss and extend the station’s working time.

**Figure 2:**
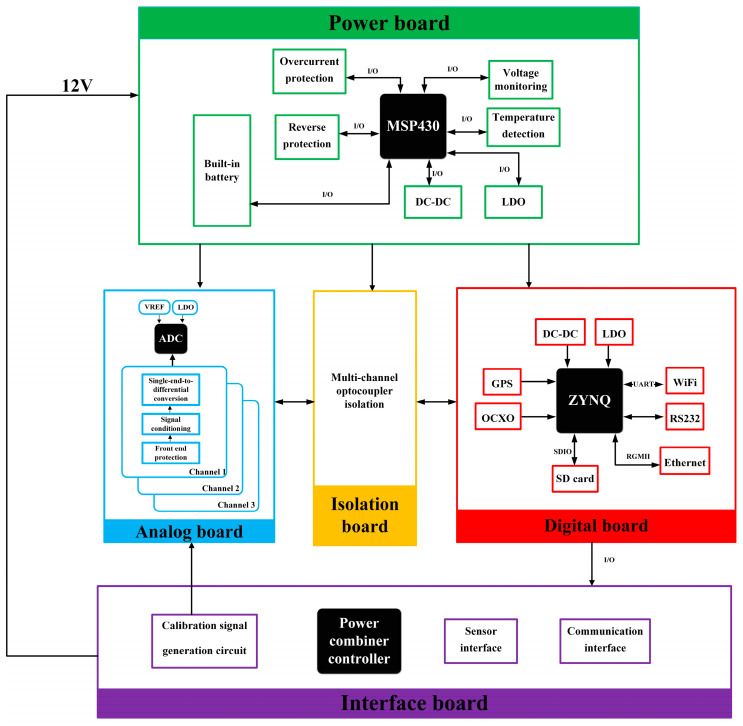
The overall architecture of the Zynq-based seismic acquisition station for polar applications. LDO: low dropout regulator; DC-DC: direct current–direct current converter; GPS: global positioning system; OCXO: oven-controlled crystal oscillator; ADC: analog-to-digital converter.

Figure 2 presents the block diagram of the seismic acquisition station designed for Antarctic subglacial lakes. The hardware circuit comprises digital, analog, interface, power, and isolation boards. These components relate to an external lithium battery, geophones, and a thermal insulation container to form a complete system.

The interface board primarily functions to connect various connectors to the circuit boards and manage the power supply of backup lithium batteries and external batteries. The power board, based on an MSP430 microcontroller, is responsible for the power management, power control, and status monitoring of each component of the seismic acquisition station. The analog board was designed with three channels and is responsible for filtering and amplifying the output analog signals from sensors. It utilizes an ADS1274 24-bit Δ-Σ ADC for high-precision analog-to-digital conversion. The digital board incorporates a Zynq series SoC (system-on-chip), integrating an FPGA and ARM to manage the storage of acquired data (on an SD card), as well as to allow for Wi-Fi wireless human–machine interaction and high-speed Ethernet data transmission. The isolation board employs isolation chips to minimize interference between analog (seismic acquisition) and digital control boards. The circuit boards are interconnected using high-reliability connectors and cables to facilitate power and signal transmission.

### 2.2. Key Technologies

#### 2.2.1. Zynq-Based High-Speed, Real-Time Data Processing

*Hardware design of Zynq*

**Figure 3:**
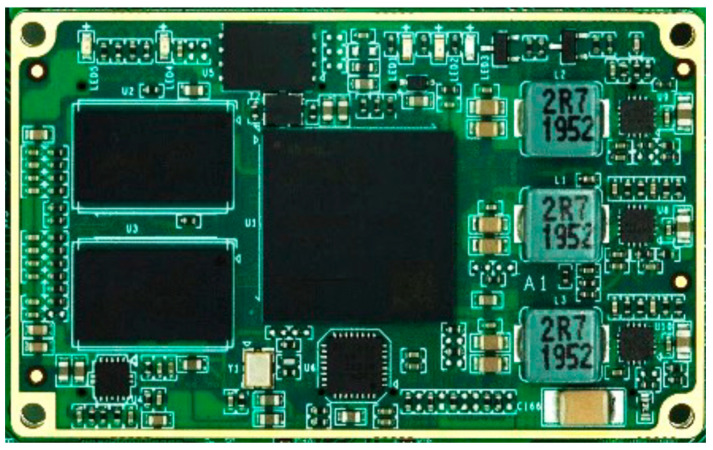
Image of the SoC.

To achieve remote data quality monitoring and high-speed, real-time data recycling, the digital control board utilizes an SOM-TLZ7020 industrial-grade (lowest operating temperature: −40 °C) SoC integrated with a Xilinx Zynq 7000 series XC7Z020 heterogeneous multi-core high-performance low-power processor. The SoC integrates a dual-core ARM Cortex A9 processing system module (PS) and an Artix 7 architecture 28 nm programmable logic module (PL). Equipped with a 512 MByte double data rate 3 (DDR3) random access memory (RAM), a 4 GByte embedded MultiMediaCard (eMMC), and a 256 Mbit quad serial peripheral interface (QSPI) Nor Flash, the SoC ensures efficient operation. The core board, an immersion gold 12-layer board, utilizes four 80-pin B2B connectors to expose all available pins of the PL and PS, thus fulfilling the requirements of various application scenarios [21]. An image of the SoC is depicted in Figure 3.

The PS of the XC7Z020 has a maximum frequency of 766 MHz and supports interfaces such as gigabit Ethernet, universal serial bus (USB), a controller area network (CAN) bus, secure digital input/output (SDIO), a serial peripheral interface (SPI), a universal asynchronous receiver–transmitter (UART), an inter-integrated circuit (I2C), and general-purpose input/output (GPIO). On the other hand, the PL has 85K logic cells, 106K registers, 4.9 Mbit Block RAM, 220 multipliers, four clock management units, 16 global clock networks, six user input/output (I/O) banks, and a maximum of 253 user I/Os [22]. Communication between the PS and PL is facilitated by an internal high-performance bus.

**Figure 4:**
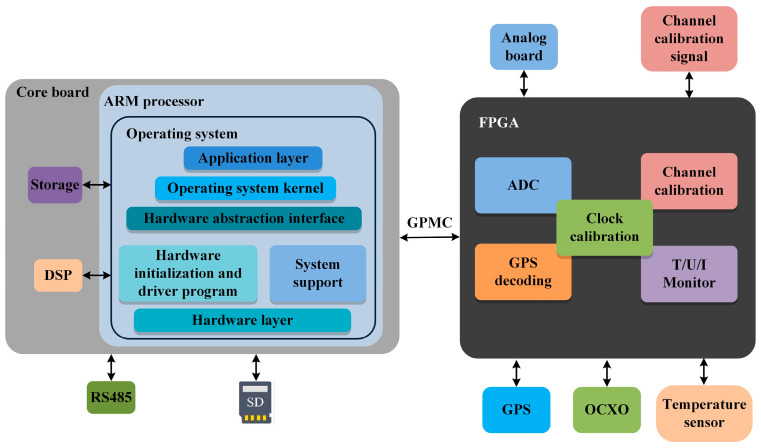
Traditional FPGA-ARM architecture for digital controllers.

Traditional digital control schemes often employ a dual-chip design (Figure 4). The FPGA is connected to the ARM, the main control unit, via a parallel general-purpose memory controller (GPMC) bus. The FPGA is responsible for high-speed data collection and processing, incorporating functional modules such as oven-controlled crystal oscillator calibration, ADC data acquisition, channel calibration, and the monitoring of temperature, current, and voltage (T/I/U) measurements, as well as global positioning system (GPS) message reception and decoding. Meanwhile, the ARM operates with an embedded operating system for system control and human–machine interaction. Data transmission between the ARM and FPGA is realized via a GPMC bus for unified interfacing. The GMPC bus includes eight chip select lines, with CS2-CS7 designated for communication with the FPGA and CS0-CS1 used for flash memory. Data transmission between the GPMC bus and the FPGA utilizes a chip select addressing mechanism. Specifically, the processor first determines whether the accessed address is within the register’s addressing range. If the physical address falls within this range, the address information undergoes decoding. This decoding process involves an AND operation between the address and the CONFIG7 address mask to derive a new address. If this new address matches the base address of CONFIG7, the corresponding chip select pin is set to a low level.

**Figure 5:**
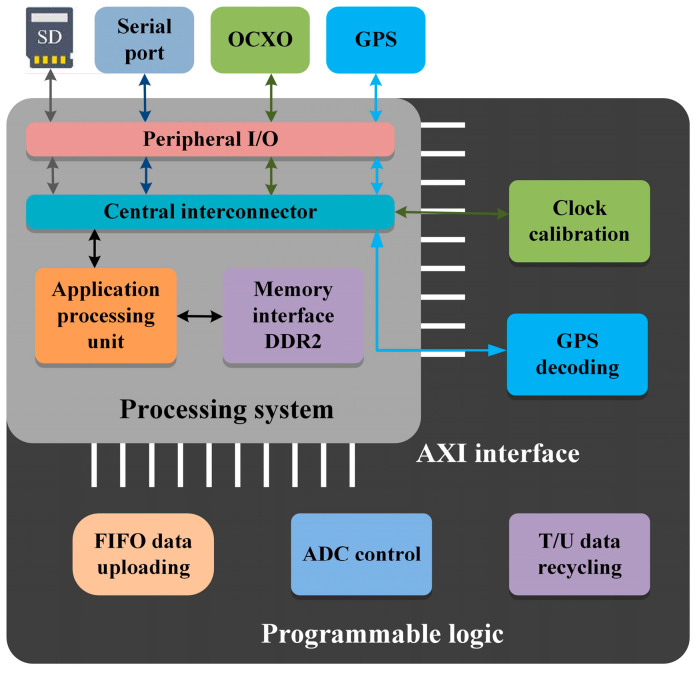
Structural chart of Zynq design.

Compared with the traditional dual-chip design utilizing a GPMC bus for data transmission between the ARM and FPGA, Zynq employs the Advanced eXtensible Interface (AXI) bus protocol, featuring independent channels for improved efficiency, faster data transmission, and a higher frequency, enabling hardware-level acceleration. The structure chart of the Zynq design is depicted in Figure 5. Additionally, Zynq is equipped with a 256 KB high-speed scratchpad memory, a shared memory accessible by both the PS and PL. In the data transmission process of the seismic acquisition station, the PS and PL utilize a central interconnector for unified peripheral deployment. The PS primarily manages system control functions, and the PL manages data collection, computation, and processing for the sub-modules. The PS transfers the control commands of the working parameter packet to the PL via the AXI bus protocol, significantly enhancing the data transfer speed and overall system performance. The 64-bit AXI port theoretically offers a bandwidth of up to 1.067 Gbps, and after testing and optimization, the actual read and write speed could reach up to 1.016 Gbps [23].

*Programming of Zynq*

This subsection explains the FPGA Verilog hardware description language (HDL) programming for the PL of Zynq and the embedded Linux system programming for the PS of Zynq. These enable the control board to manage overall data acquisition, data fusion and processing, GPS positioning, system clock synchronization and calibration, and station networking.

The programming of Zynq on the control board was completed in the Vivado 2017.4 integrated design and development environment [24]. This environment facilitated the convenient development and interfacing of both the PL and PS and allowed for the flexible integration of various IP cores, accelerating software design. The Zynq software comprised two parts: the FPGA program for the PL and the embedded Linux program for the PS. The PL software was written in Verilog HDL, adopting a top-down software design approach. Functionally, it was divided into ADC data acquisition, GPS message reception and decoding, and system clock calibration and synchronization modules. Verilog HDL files were written for each function, focusing on key signal detection, data flow, processing, storage, and the output. Each functional module had input and output interfaces. In the top-level file, these functions were instantiated, and interfaces and data interactions between modules were established. After logical synthesis and a timing simulation, the program was ready for configuration download and board-level debugging.

**Figure 6:**
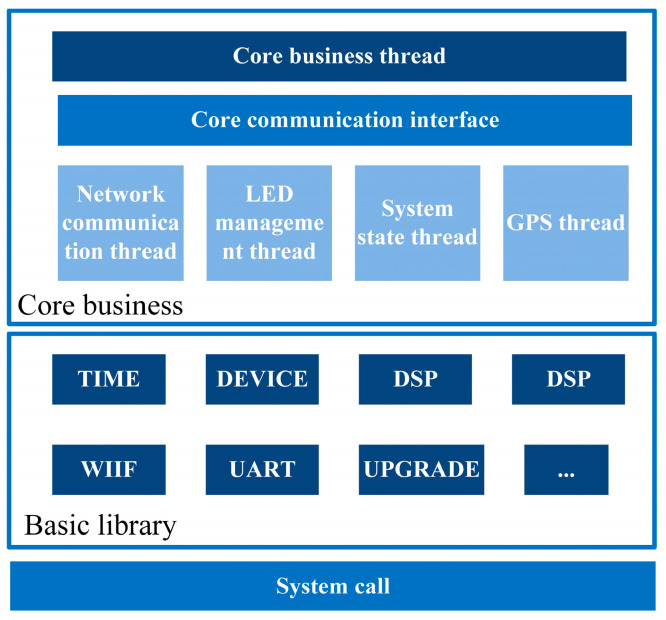
Architecture of Zynq software design.

The embedded software of the seismic acquisition station runs on a Linux platform and is responsible for system resource scheduling, management, and data storage. A top-down, functional, and modular software design approach was adopted, and the software architecture consists of a main thread and several sub-threads (Figure 6). The software is divided into three main sections: the core business, basic library, and system call. The core business of the seismic acquisition station consists of one main thread and four mutually independent sub-threads. The main thread is primarily responsible for initialization, sub-thread activation, data acquisition, and instrument calibration. The network communication thread manages self-storage, wired/wireless network construction, and communication with the master station. The light-emitting diode (LED) management thread controls basic human–machine interactions via LED indicators. The system state thread receives and analyzes voltage and temperature information uploaded from the power board, evaluates the remaining capacity of the control board’s SD card, and manages the collection, uploading, and processing of station statuses, such as the number of satellites. The GPS thread reads the GPS time information parsed by the PL, updates the GPS status display, and determines whether secondary positioning is necessary. The basic library of the system provides fundamental platform support for device hardware control functions. Both threads and processes can directly call interface functions to execute corresponding functions. The basic library mainly includes functions that rely on the underlying operating system and system calls from the GNU C Library, such as Wi-Fi management, UART management, device management, net management, disk management, upgrades, and time management interfaces. The system call section provides an abstract interface for the hardware by adding an intermediate layer between the user space process and the hardware, ensuring system stability and security by preventing the application from managing disk types and media. In the Linux system, system calls are the only means for user space processes to access the kernel, except for exceptions and interruptions.

#### 2.2.2. Design of Multi-Channel Data Acquisition Circuit

**Figure 7:**
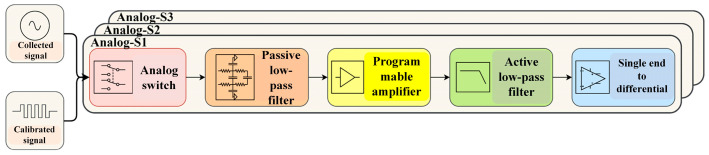
Block diagram of the data acquisition board.

The block diagram of the data acquisition channel circuit is illustrated in Figure 7. We designed three channels for each acquisition station. Analog switches were employed to toggle between calibrated signals and signals acquired by the geophone. The seismic acquisition station can switch between acquisition mode and calibration mode through software control. Subsequently, the signals pass through a resistor–capacitor (RC) passive low-pass filter, a programmable amplifier, a second-order Sallen–Key (SK) active low-pass filter, and a single-ended-to-differential conditioning circuit before entering the ADS1274 analog-to-digital converter for analog-to-digital conversion. Finally, the digital signals are transferred to the Zynq digital controller for further processing.

#### 2.2.3. Temperature Drift Compensation for ADC

A neural network [25] was employed for temperature drift correction to ensure the data acquisition accuracy of the ADS1274 ADC in low-temperature environments. Referring to the temperature drift correction of a gyroscope [26], it is believed that a similar approach could be applied to correct the temperature drift of the high-precision ADC in extreme temperature conditions.

By adjusting the temperature in a cryogenic chamber, different environmental conditions were simulated, allowing the ADC chip to collect data on different voltages at various temperatures. After cleaning, balancing, and augmenting the data, a training dataset was established and used to train a multi-layer perceptron (MLP) model that aligns with the characteristics of the chip.

**Figure 8:**
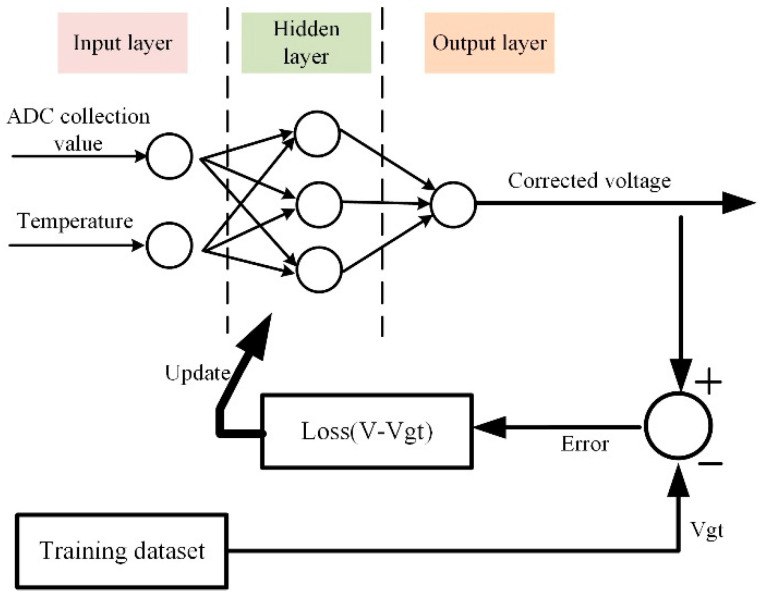
Neural network training setup.

As illustrated in Figure 8, the neural network model was trained using the aforementioned training set and the “Post-Correcting ADC Errors with Neural Networks” model architecture and training method proposed by the Dutch company NXP [27], thereby learning and simulating the behavior and output of the ADC chip at different temperatures to realize temperature drift compensation. The model adopts a basic MLP structure comprising an input layer (collection values and temperature values), an output layer (corrected voltage), and a hidden layer, using voltage deviation as the loss function. The model was implemented using PyTorch [28], and the pre-collected and cleaned data were fed to the model for training.

The model features two deployment options: data analysis and edge computing. If the model is deployed at the data analysis step, the prediction method of the model is integrated into the data analysis host computer. The slave computer collects additional temperature data and stores them on an SD card. The model is applied during the data decoding process in the host computer for temperature correction. Data analysis deployment is easier to develop than edge computing deployment, but the former may require a longer data analysis time than the latter.

If the model is deployed at the edge computing step, the model is quantized and deployed to the FPGA via the FPGA inference neural network. Temperature correction is performed during the data acquisition and storage stages. This deployment scheme is more efficient than data analysis deployment but may compromise model accuracy due to quantization and consumes the FPGA’s look-up table resources.

After considering these factors comprehensively, the data analysis deployment option was selected.

#### 2.2.4. Low Power Consumption Design

*Hardware design for power board*

**Figure 9:**
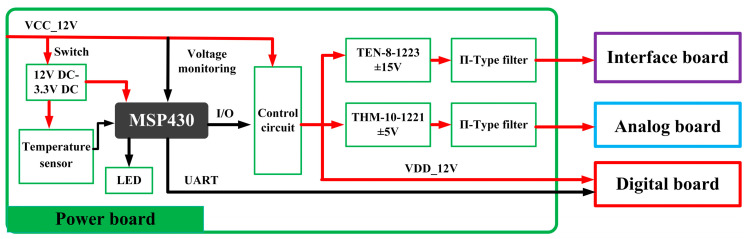
Design of power board.

The primary function of the power board is to ensure a stable and reliable power supply to the circuitry, meeting the required power supply parameters of the system. Additionally, it considers the seismic acquisition station’s overall low-power design to avoid unnecessary power loss and extend the instrument’s operational time. The board mainly implements functions such as direct current to direct current (DC-DC) conversion, power-on control, power supply state identification, voltage monitoring, temperature monitoring, LED indicator control, and serial data transmission. The main controller of the power board utilizes a 16-bit ultra-low-power MSP430G2553 microcontroller [29], known for its rich internal resources, easy development, and high cost-effectiveness, fulfilling the requirements of this design. The design scheme of the power board is depicted in Figure 9.

*Software design for power board*

This C language program was based on the MSP430 for power management, temperature monitoring, and the LED status display.

The total active power consumption and standby power consumption of the seismic acquisition station were tested and found to be 6.607 W and 0.024 W, respectively. Due to this significant difference, it was necessary to design a power management system specifically for the seismic electromagnetic acquisition station.

The major functions of the power board include power-on control, power supply state identification, voltage monitoring, temperature monitoring, LED indicator control, and serial data communication. The source power and temperature data are collected, converted to voltage and temperature information, and sent to the control board to reduce the data processing load on Zynq. The power board manages all power and temperature monitoring tasks and protects the overall circuit by cutting off power when the temperature or voltage exceeds the threshold. Upon powering up the device, only the 3.3 V microcontroller is activated to initialize configurations for functional registers such as the clock, GPIO, timer, ADC, UART, and comparator. Subsequently, the power board is initialized, the main control board’s input power is turned on, the acquisition board’s power is turned off, and the current power status, voltage, and temperature information are uploaded. Based on the control board’s command, the acquisition board’s power is turned on. This approach allows for controlling the power sequence of the seismic acquisition station system through software, ensuring a logical power-up sequence and safe power supply.

**Figure 10:**
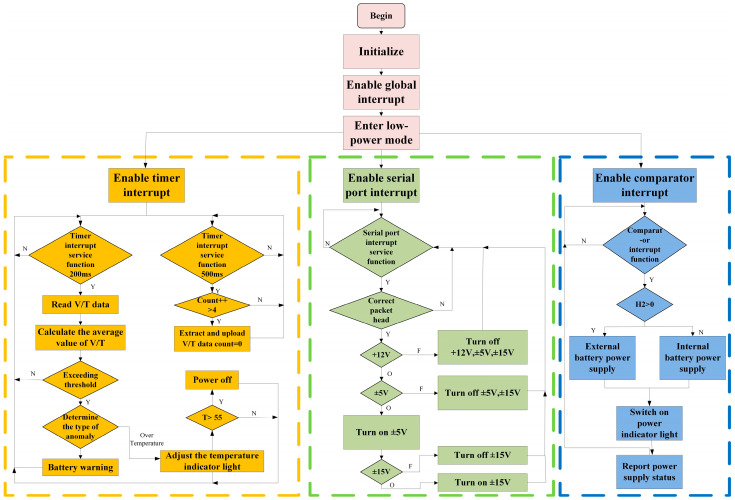
Flowchart of MSP430 microcontroller.

The power board uses an ultra-low-power mixed-signal microcontroller, MSP430G2553, as the main controller, which was programmed for power management using CCS11.0.0. The program flowchart is shown in Figure 10.

#### 2.2.5. Design of Upper Computer Software

**Figure 11:**
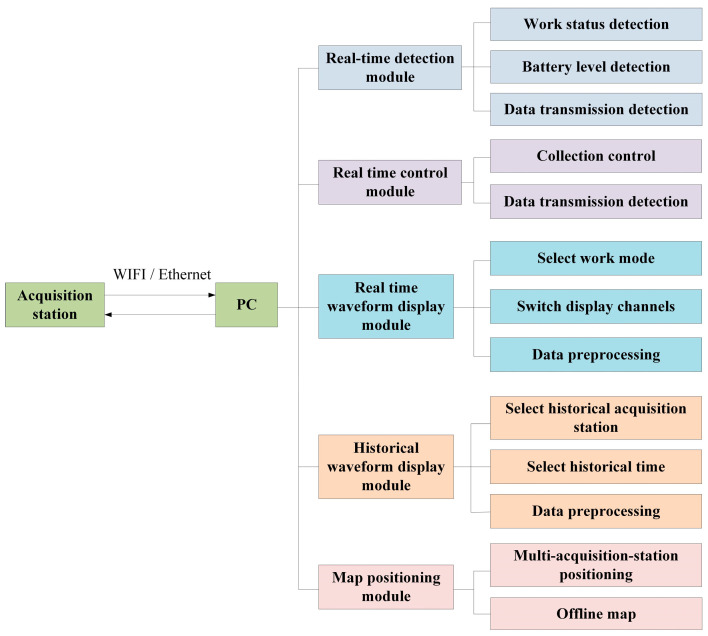
Block diagram of PC-end software.

**Figure 12:**
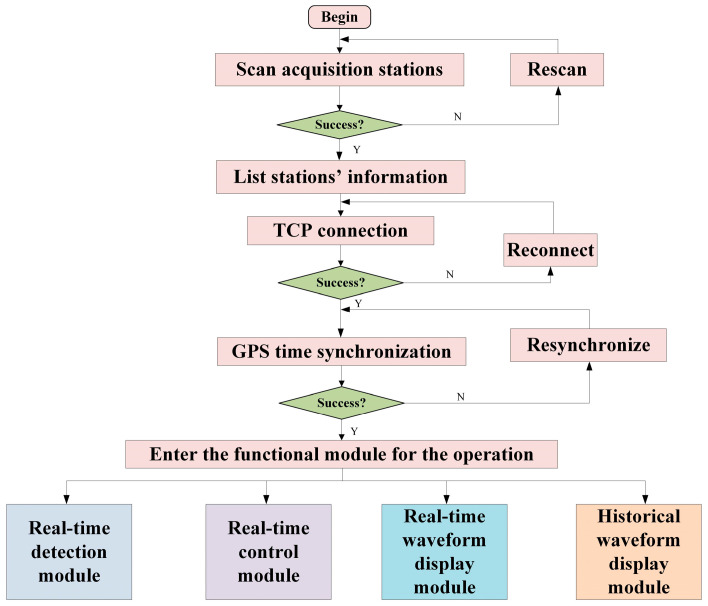
Workflow of PC-end software.

**Figure 13:**
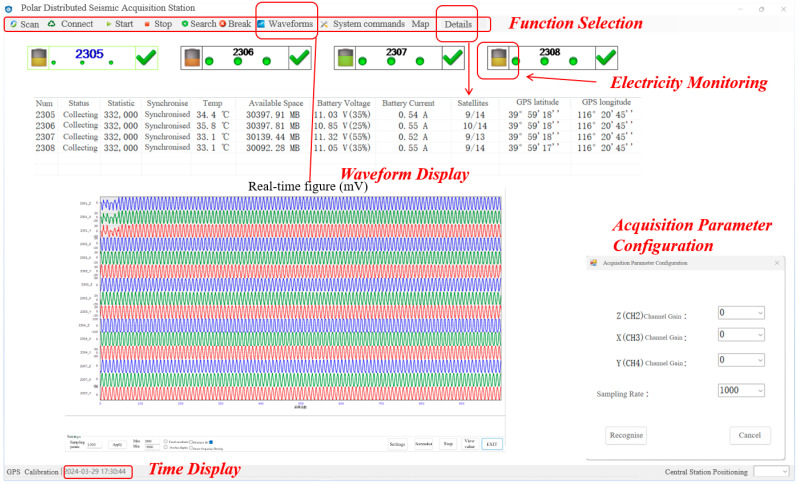
The user interface of the PC-end software.

The data transfer and storage system supports wired and wireless data transmission modes by combining a user datagram protocol (UDP) and transmission control protocol (TCP). Wired data transmission employs Ethernet for data transfer, and wireless data transmission utilizes a self-built 2.4 GHz wireless local area network, eliminating the need for network operator services. The personal computer (PC)-end software was developed using Python [30] and is responsible for the real-time monitoring of the seismic acquisition station’s status, real-time batch control, real-time waveform display, historical waveform playback, and data preprocessing. The functional architecture of the software control system is illustrated in Figure 11, and the flowchart for the PC-end main program is presented in Figure 12. The user interface is shown in Figure 13. In the field, the user simply needs to turn on the power switch on the acquisition station and set up the geophones. Once powered on, GPS synchronization will occur automatically. To use the system, the user first clicks the “Scan” button in the upper left corner of Figure 13 to scan for powered-on acquisition stations. After scanning, the user clicks “Connect” to connect to the selected station. Once connected, detailed information such as the station’s temperature, synchronization status, location, and storage space can be viewed in the “Detail” section. In the “System Commands” section, the user can configure acquisition and display parameters. Finally, the user can start data acquisition by clicking “Start.” The host software allows for the real-time monitoring of the waveforms.

## 3. Results and Discussion

### 3.1. Power Consumption Testing

The actual application capabilities of the developed seismic station needed to be tested. In the laboratory, using a signal generator, the performance of the seismic acquisition station was assessed via background power consumption testing, noise testing, dynamic range testing, channel crosstalk testing, and synchronization accuracy testing.

We conducted power consumption tests on the developed acquisition station, and the results are shown in Table 1. The results indicate that the total power consumption during operation is 6.646 W, while the standby power consumption is 0.021 W. The significant difference between these two values highlights the necessity of designing a power management system for the acquisition station.

The system utilizes a 12 V lithium battery, with the operational duration determined by the battery’s capacity. Our acquisition station is used in conjunction with an electromagnetic-controlled seismic source for seismic exploration, ensuring no environmental damage or pollution. The system does not involve any disposable equipment, and after measurements are completed, the acquisition station and battery components are brought back, leaving no waste behind. Therefore, there are no issues regarding material handling or environmental pollution.

### 3.2. Background Noise Testing

The background noise of the acquisition system is an important indicator for measuring system accuracy, which characterizes the ability of the acquisition system to receive weak signals. Specifically, we connected the input terminals of each channel interface of the acquisition station, set different sampling rates, and set different channel gains. After collecting noise data over a period of time, the collected data were processed using MATLAB to calculate the equivalent input noise effective value shown in Table 2. The calculation of the equivalent input noise peak voltage can be expressed using the standard deviation, as shown in (1):

(1) $$V_{noise}=\frac{\sqrt{\frac{1}{N}\sum_{i=0}^{N}{\left(V_{i}-\bar{V}\right)}^{2}}}{Gain}$$

### 3.3. Dynamic Range Testing

The dynamic range of the acquisition station is a measure of the upper and lower voltage ranges of the signal that can be collected, which corresponds to the effective value of the background noise mentioned above. In order to test the dynamic range of our proposed acquisition system, we set the gain of the acquisition channel to one and input the standard sine wave signal generated by the signal generator into the acquisition channel. We continuously adjusted the signal amplitude until saturation occurred, and the signal amplitude was the effective value of the upper limit maximum unsaturated voltage. According to (2), the dynamic range of the seismic channel could be calculated as 130 dB; the results are shown below in Table 3.

(2) $$DR=20\log_{10}\frac{V_{\max}}{V_{\min}}$$

where $V_{\max}$ is the maximum signal voltage that the circuit can handle, and $V_{\min}$ is the minimum signal voltage that the circuit can handle. The minimum signal voltage is usually the smallest effective signal voltage that the circuit can detect, generally limited by the noise level.

### 3.4. Synchronization Accuracy Testing

Each acquisition station is synchronized with GPS second pulses separately to achieve multi-station synchronization. Additionally, each acquisition station outputs a local PPS (Pulse Per Second) signal based on the constant-temperature crystal oscillator frequency as an indicator of local time updates and the completion of synchronization at the acquisition station. Therefore, by comparing local second pulses between acquisition stations, we can measure the synchronization accuracy between different acquisition stations.

**Figure 14:**
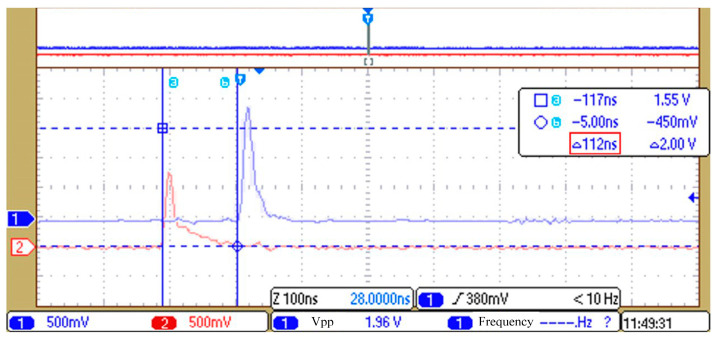
Results of synchronization accuracy between acquisition stations (through color reversal only).

As shown in Figure 14, channels 1 and 2 are connected to local PPS signals from different acquisition stations. The synchronization interval of the two PPS signals was 112 ns. After repeating measurements multiple times, the average synchronization interval of the two PPS signals was around 110 ns.

The analysis of the sources of this error showed that it mainly came from the error of GPS chips receiving second pulses at various acquisition stations, the inevitable quantization error of using 12.288 MHz counting during crystal oscillator calibration, and the time error of using an oscilloscope to capture local second pulses on the wiring. The synchronization accuracy can meet the requirement of a maximum sampling rate of 8k SPS.

Moreover, we tested other performances, and a comparison of the main performance indexes of the seismic acquisition station developed in this study and those of other popular ones are presented in Table 4.

### 3.5. Outdoor Testing

**Figure 15:**
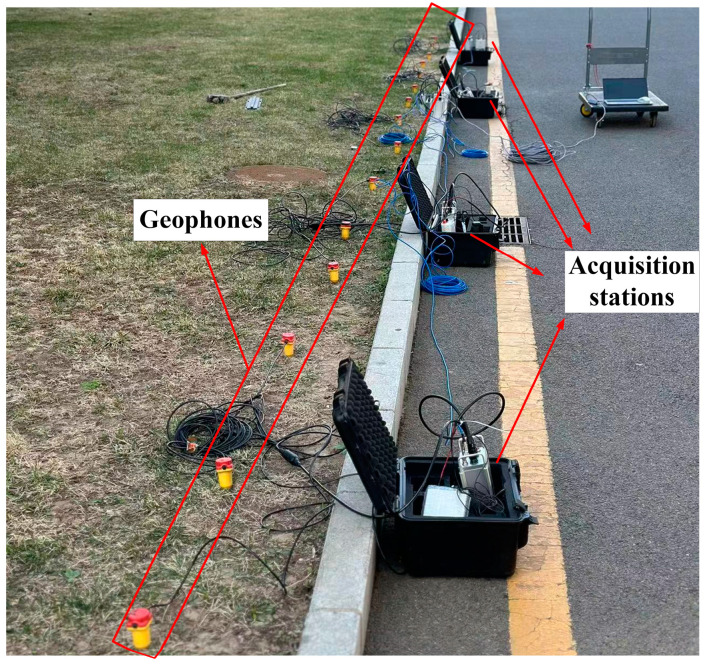
Image of outdoor iron hammer source test.

**Figure 16:**
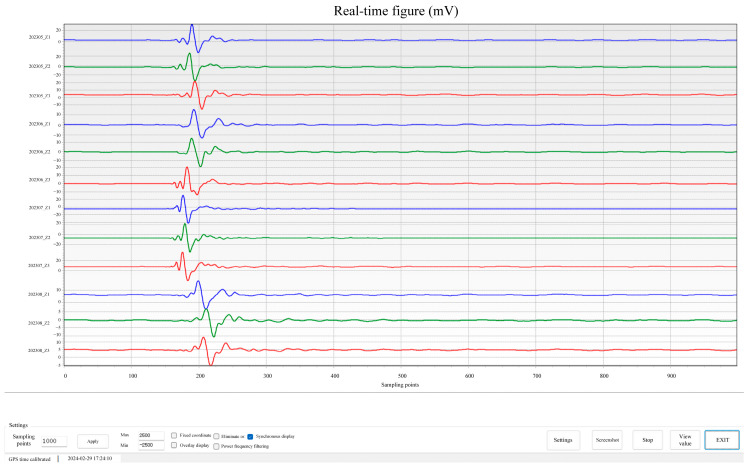
Results of the outdoor iron hammer source test.

An outdoor test was performed using an iron hammer as an artificial seismic source to further validate the stability and feasibility of the developed seismic acquisition station. Figure 15 presents an image of the test setup on campus. Several seismic stations were arranged to collect seismic signals stimulated by striking an iron block with an iron hammer. The results displayed on the master computer are shown in Figure 16, indicating noticeable waveform fluctuations during the strikes and consistent channel performance.

**Figure 17:**
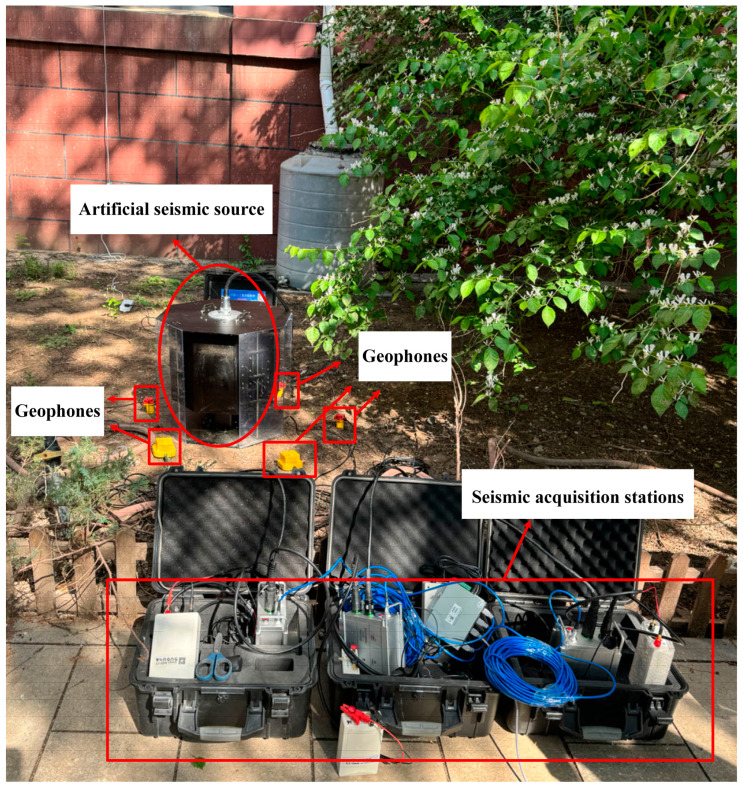
Image of outdoor artificial seismic source test.

**Figure 18:**
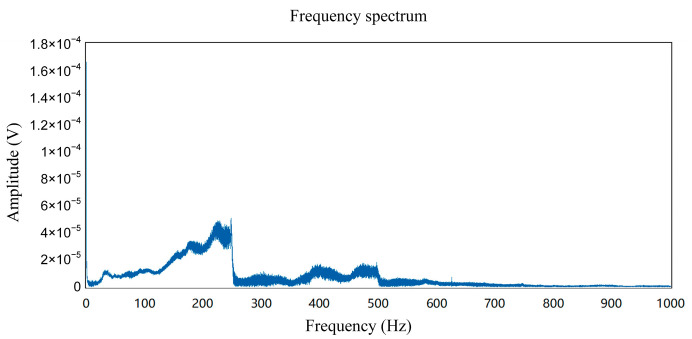
FFT results of the outdoor artificial seismic source test.

We conducted frequency sweep and fixed frequency tests using an artificial seismic source ranging from 2 Hz to 250 Hz, as shown in Figure 17. Three seismic acquisition stations collected data around the artificial seismic source. The results of the sweep frequency test were analyzed using a frequency spectrum to obtain Figure 18, indicating that the acquisition stations successfully collected the transmitted signals ranging from 2 Hz to 250 Hz.

**Figure 19:**
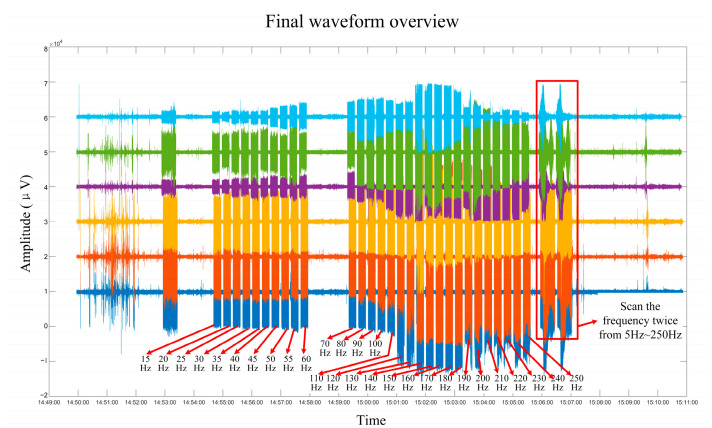
Final waveform overview of artificial seismic source test.

**Figure 20:**
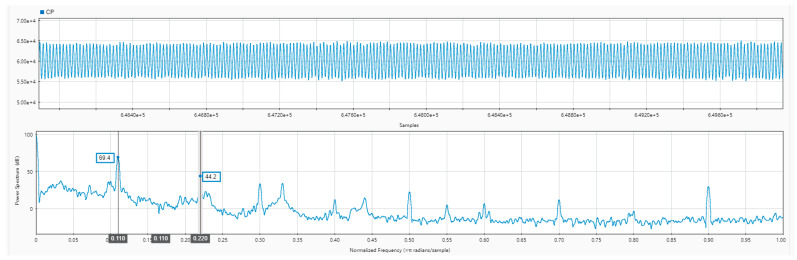
The 55 Hz power spectrum of the artificial seismic source test.

During the 15 Hz–250 Hz fixed frequency test, each fixed frequency time was 15 s with an interval of 5 s. The final waveform overview is shown in Figure 19, and the 55 Hz fixed frequency signal was extracted. After analyzing the power spectrum of the received signal at the acquisition station, as shown in Figure 20, we found that this system can accurately collect artificial seismic source signals.

### 3.6. Field Test in the Antarctic

**Figure 21:**
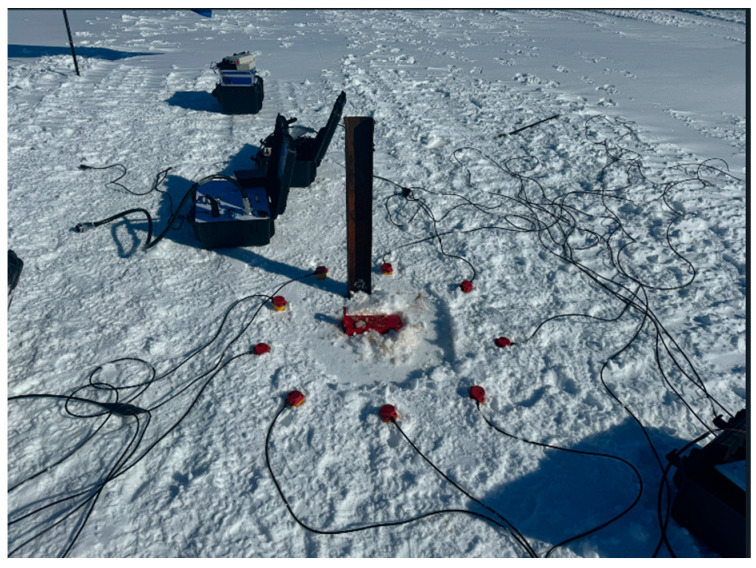
Image of a field test in the Antarctic.

Ultimately, a field test was conducted in the Antarctic (coordinates: 76.38421118E, 69.58561245N; altitude = 671.0 m) on 9 January 2024 (UTC 09:54:24). As illustrated in Figure 21, three seismic acquisition stations were tested by arranging nine geophones in a circular pattern with a diameter of 1.20 m and a spacing of 0.4 m. The seismic source was simulated by striking an iron block. Data were successfully recorded.

**Figure 22:**
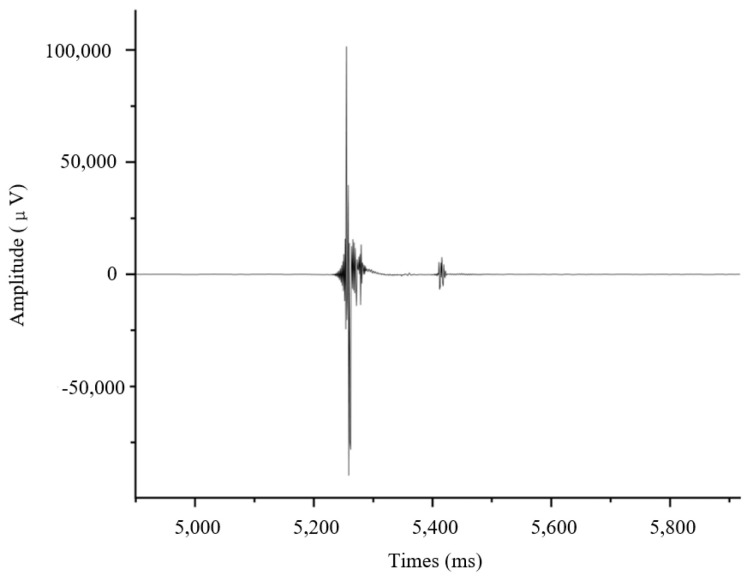
First-arrival picking of the reflected wave.

The first-arrival picking results are shown in Figure 22. The interval between the direct and reflected waves on the seismogram was 169 data points, corresponding to a time interval of 338 ms, given the 2k SPS sampling rate. With the known propagation velocity of longitudinal waves in the ice layer being 3230 m/s, this time interval shows that the round trip propagation time of the seismic wave is equivalent to twice the length of the propagation path at a vertical incidence or twice the thickness of the ice layer. The calculated ice layer thickness was approximately 545.9 m. The real ice layer thickness was 545 m according to China’s 40th Antarctic scientific expedition, consistent with the result.

## 4. Conclusions

This study developed a seismic station based on the Zynq platform to fulfill the seismic data acquisition requirements in the extreme Antarctic environment. This system can effectively acquire multi-channel seismic data in the harsh low-temperature conditions in the Antarctic region and features remote data quality monitoring capabilities and high-speed real-time data transmission. Its notable characteristics include a compact design, high-precision data collection, excellent cold tolerance, low power consumption, wireless communication, high system synchronization accuracy, and real-time control functionality. This study discusses the seismic station’s software architecture and hardware components and comprehensively evaluates the system’s performance. Validating the reliability and practicality of the system in extreme conditions comprised testing conducted not only in laboratory and outdoor settings but also in the Antarctic by using specially designed geophones, batteries, and thermal containers. The test results confirmed that the system was stable, easy to use, and entirely fulfilled the high standards required for polar exploration, providing valuable technical support for scientific research in the Antarctic. However, there are still some limitations that need to be addressed. The limitations of this study and future research directions are as follows.

In polar environments, energy supply is extremely limited. Although we designed a low-power system, battery endurance and energy management remain critical issues during long-term field operations, especially since battery performance can significantly degrade in extreme cold conditions. To address these challenges, future improvements will include adopting more efficient batteries and advanced energy management systems. For instance, we plan to introduce a lithium ion battery heating system to ensure good battery performance under low-temperature conditions. Additionally, we will explore and develop solutions using renewable energy sources such as solar power to supplement battery power and extend the operation time of the equipment. Specifically, integrating and optimizing solar panels will be an important research direction, aiming to enhance their energy conversion efficiency and durability in polar environments. Through these improvements, we expect to significantly enhance the system’s energy management capabilities in polar environments, ensuring stable operation during long-term fieldwork.

Conducting more field tests in polar environments will verify the system’s broad applicability and collect more data for analysis and improvement. By incorporating it in actual environmental applications, we can further validate and improve the system’s performance and reliability. Currently, preparations are underway for the second Antarctic expedition of the acquisition station, scheduled for testing in Antarctica next year. In this test, we will focus on evaluating the system’s stability and data acquisition capabilities under extreme cold and harsh weather conditions. Additionally, we will collaborate with local research teams to conduct joint experiments to comprehensively assess the system’s field application effectiveness. Through these field tests and applications, we aim to identify and address potential issues encountered during actual use, further optimizing the system design and enhancing its practicality and reliability in polar environments.

Furthermore, multidisciplinary collaboration is an important strategy to address the complexity and variability of polar environments. We plan to closely cooperate with experts in glaciology and geology to comprehensively understand and solve the technical and scientific challenges in polar exploration. By collaborating with glaciology experts, we can study the impact of glacier movement on seismic wave propagation characteristics in depth, thereby improving data accuracy and interpretive capabilities and optimizing the selection and deployment strategies of seismic signal acquisition points. The involvement of geology experts will help us gain a deeper understanding of the polar subsurface structure, optimize seismic data acquisition and processing methods, and potentially reveal new geological phenomena and resources. By integrating the expertise and technologies of glaciology and geology, we aim to comprehensively enhance the performance and adaptability of the polar seismic acquisition station, providing a solid technical foundation and broad research prospects for future polar scientific research.

## Figures and Tables

**Table 1:** Measured power consumption results of the circuit boards.

Power Board + Interface Board	Main Control Board	Acquisition Board
0.021 W	5.624 W	1.001 W

**Table 2:** Root mean square voltage of background noise for each channel.

Sampling Rate/SPS	Gain	Channel 1/μV	Channel 2/μV	Channel 3/μV
1k	1	5.5924	5.6091	5.6137
	10	0.6358	0.6474	0.6494
	100	0.3013	0.2902	0.3078
	1000	0.2840	0.2688	0.2816
2k	1	5.6892	5.8180	5.8063
	10	0.7007	0.7033	0.7063
	100	0.3935	0.3848	0.3941
	1000	0.3743	0.3711	0.3820
4k	1	6.0020	5.9929	5.9515
	10	0.8202	0.8071	0.8184
	100	0.5437	0.5441	0.5403
	1000	0.5083	0.5061	0.5294
8k	1	5.9748	5.9028	5.9370
	10	0.8251	0.8085	0.8263
	100	0.5479	0.5327	0.5435
	1000	0.5188	0.5008	0.5302

**Table 3:** Results of dynamic range tests.

Sampling Rate/SPS	Channel	Dynamic Range/dB
1k	Channel 1	135.88
	Channel 2	136.36
	Channel 3	135.83
2k	Channel 1	133.48
	Channel 2	133.57
	Channel 3	133.39
4k	Channel 1	130.80
	Channel 2	130.82
	Channel 3	130.53
8k	Channel 1	130.71
	Channel 2	130.79
	Channel 3	130.46

**Table 4:** Results of seismic station performance tests.

Index	Developed Acquisition Station	SUMMIT X One	GCL/GSX/GSB	SmartSolo
Dynamic range	>130 dB	132 dB	124 dB	145 dB
System synchronization accuracy	112 ns	/	1 μs	10 μs
Channel gain	0–60 dB	0–40 dB	0–36 dB	0–36 dB
Number of channels	3	1	1/3/3	1
Data transmission/save method	Wireless or wired or local storage	Wired	Wireless or local storage	Local storage
Operation temperature	−50 °C to +70 °C	−25 °C to +60 °C	−40 °C to +60 °C/ −40 °C to +85 °C/ −40 °C to +60 °C	−40 °C to +70 °C

## Data Availability

Our research is supported by national projects; thus, the data are not publicly accessible due to a confidentiality agreement.
